# Outbreak of equid herpesvirus 1 abortions at the Arabian stud in Poland

**DOI:** 10.1186/s12917-020-02586-y

**Published:** 2020-10-06

**Authors:** Karol Stasiak, Magdalena Dunowska, Jerzy Rola

**Affiliations:** 1 Department of Virology, National Veterinary Research Institute, Al. Partyzantow 57, 24-100 Pulawy, Poland; 2 Institute of Veterinary, Animal and Biomedical Sciences, Massey University, Palmerston, North, New Zealand

**Keywords:** Abortions, Equid herpesvirus 1, EHV-1, Latency, ORF30, ORF68, PCR

## Abstract

**Background:**

Equid herpesvirus 1 (EHV-1) infections are endemic worldwide, including Poland. Many are subclinical, but some are associated with respiratory disease, abortion, neonatal foal death, or neurological disease. We describe an outbreak of abortions in Arabian mares at a well-managed State stud farm in Poland.

**Case presentation:**

Eight of 30 pregnant mares aborted and one gave birth to a weak foal that died within 72 h after birth. EHV-1 was isolated from all fetuses as well as from the diseased foal. All viruses belonged to the N_752_ variant based on the predicted open reading frame (ORF) 30 amino acid sequence. All were identical to each other and to previous EHV-1 viruses from the same stud based on the ORF68 sequence analysis. The outbreak coincided with the lapse in the routine yearly EHV-1/4 vaccinations of the mares.

**Conclusions:**

Multiple abortion due to EHV-1 infection can occur in well-managed groups of horses. Reactivation of latent EHV-1 in one of the resident mares followed by a horizontal spread was considered the most likely explanation for the outbreak. Routine vaccination is an important part of a herd-heath program.

## Background

Respiratory tract infections with equid herpesvirus 1 (EHV-1) are common in horses. Although many such infections are subclinical, some can lead to serious economic losses [1]. The virus is classified in the family *Herpesviridae*, subfamily *Alphaherpesvirinae* and genus *Varicellovirus.* While sporadic cases of EHV-1 associated disease are fairly common, of most economic importance are outbreaks of abortions (“abortion storms”) [2, 3], neonatal deaths [4] or neurological disease [5, 6] with multiple horses on one property affected.

Following primary infection horses are believed to become latently infected with EHV-1 for life [1]. During latency, viral genome is maintained in the episomal form in trigeminal ganglia or in T lymphocytes, without expression of structural genes and without production of infectious viruses [7–9]. The virus can periodically reactivate from latency, which may or may not be accompanied by disease. Consequently, it is difficult, if not impossible, to maintain EHV-1 free herd, but good management and infection control practises, coupled with vaccination, can substantially reduce the prevalence of abortion and neonatal foal death, and thus economic losses due to EHV-1 infection [10].

Diagnosis of EHV-1 abortion is based on clinical history, presentation, and detection of EHV-1 in fetal/neonatal tissues [11]. Gross pathological lesions may include icterus, hepatomegaly with necrotic foci, splenomegaly, subcutaneous or pulmonary oedema, presence of straw-coloured fluid in plural and abdominal cavities, as well as petechiation in the lungs and serosal or mucosal surfaces in other tissues [12], but severity of these lesions vary and some aborted fetuses may show no obvious gross lesions [11]. Detection of the virus in fetal tissues can be accomplished by polymerase chain reaction (PCR) [13–17], virus isolation in a variety of cell lines [18] or immunochistochemistry [19]. As PCR results can be available within hours of submission of diagnostic material to the laboratory, they usually form a basis for implementation of appropriate control measures [20, 21].

The key step in the pathogenesis of EHV-1 is its ability to establish cell-associated viraemia either after primary infection or during reactivation from latency [10]. A single nucleotide substitution at position 2254 (A to G) in the open reading frame (ORF) 30 gene, corresponding to the N to D substitution at amino acid position 752 of the viral DNA polymerase, has been proposed as a genetic marker for increased neurovirulence [22], although viruses with ORF30 N_752_ have also been detected from some cases of neurological disease [23]. The ORF30 D_752_ viruses tend to establish viraemia of higher magnitude and longer duration than N_752_ viruses [24]. While the original virulent EHV-1 strain with D_752_ genotype (Ab4) was associated with development of both neurological disease and abortions, the majority of viruses obtained from abortion cases worldwide belonged to N_752_ genotype [22]. The same authors proposed that single-nucleotide polymorphisms observed in the region spanning approximately 600 bp of ORF68 may provide a molecular marker for tracking the geographical origin of EHV-1. While the utility of ORF68 for this purpose has been subsequently challenged by some authors [25, 26], it may facilitate discrimination between the outbreak virus and other EHV-1 variants that circulate locally. Recently, a multi-locus analysis of the unique long region of EHV-1 has been suggested to provide a more comprehensive method of virus typing than ORF68 sequencing [27, 28].

In the present report we describe an abortion storm due to EHV-1 infection at one of the State stud farms in Poland.

## Case presentation

### History

From February to March 2017, multiple EHV-1 abortions occurred in a well-managed group of Arabian mares at one of the Polish State Studs. At the time, there were 30 pregnant mares (aged 5 to 18 years), 14 non-pregnant mares (aged 3 to 26 years), 25 yearlings, 31 two-years-olds and 17 stallions (aged 7 to 18 years) at the stud. At the beginning of November 2016 all pregnant mares were moved to their winter accommodation. The horses were kept in individual stalls, with some turnout time together in a shared paddock during the day. Non-pregnant mares were turned out in a separate paddock. Both paddocks were separated by hedges and a 7-m wide path which prevented direct contact between two groups of mares. Young horses (yearlings and two-years-olds) were kept in a separate barn and turned out in the distant paddocks several hundred meters from where the mares were kept. The stud personnel consisted of seven to 10 grooms, each assigned to a different group of horses. Until 2015/2016 breeding season the herd health program included vaccination of all horses against equine rhinopneumonitis (Equip® EHV1, 4, Zoetis Poland), tetanus and equine influenza (ProteqFlu®-Te, MERIAL, France), according to the manufacturer’s recommendations. Despite routine vaccination, the stud had a history of occasional EHV-1 abortions. Most often, these were single abortion cases, except for 2012/13 breeding season, when four mares aborted over a period of 3 months, between January and March 2013, including mares I and F from the current outbreak. No EHV-1 abortions were recorded in 2013/2014, 2014/2015, and 2015/2016 breeding seasons. Due to change in management, none of the pregnant mares were vaccinated against rhinopenumonitis in 2016/2017 breeding season. Young horses from the stud routinely travelled to participate in a variety of sport events, as well as auction sales in Poland and abroad. These horses were kept separate from the pregnant mares at all times. In addition, all horses that returned from various events were quarantined for 2 weeks before being reintroduced to the resident population.

**Fig. 1:**
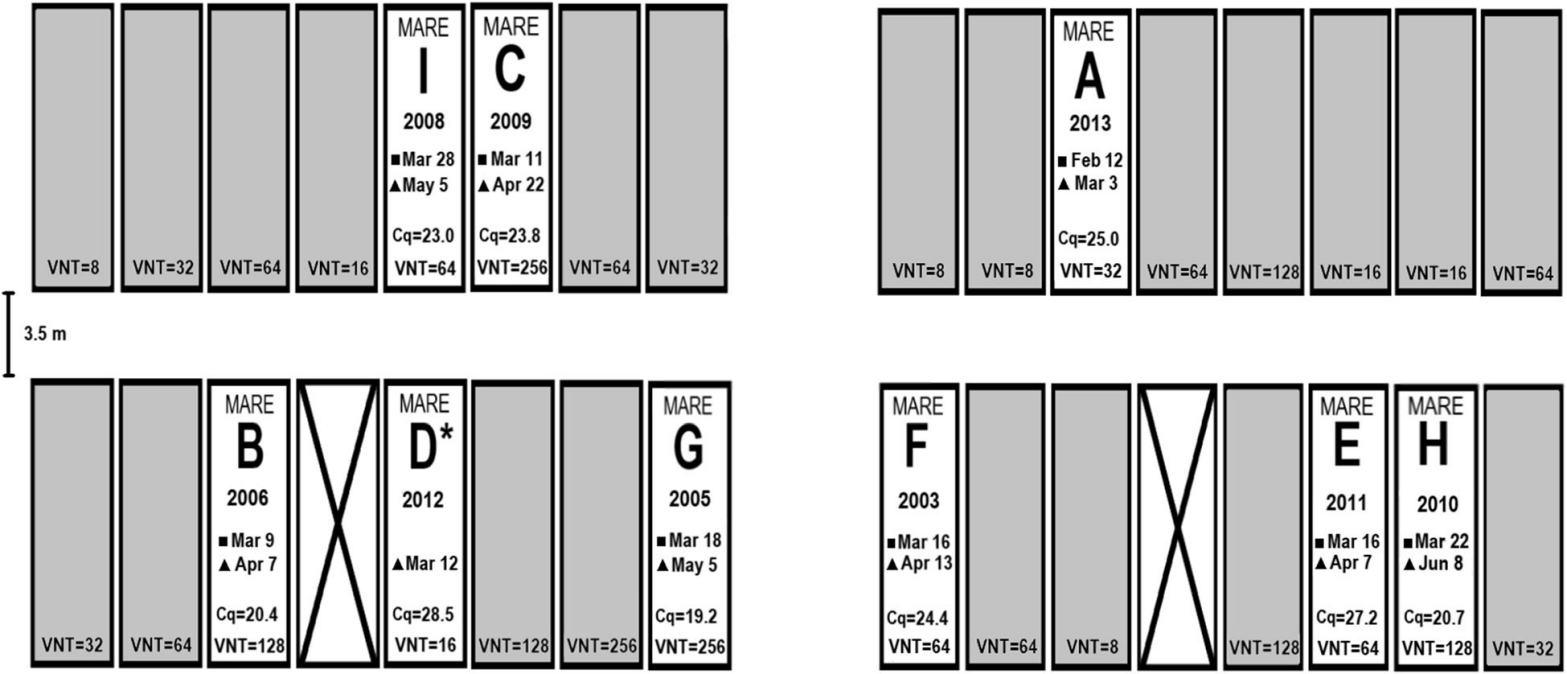
Schematic presentation of the barn with pregnant mares. Grey rectangles represent boxes with mares that did not abort in 2017. Rectangles with crossed lines represent empty boxes. Date of abortion (■), anticipated (actual for Mare D) foaling date (▲), and quantitative cycle (Cq) values obtained in EHV-1 specific qPCR with DNA extracted from pooled tissue homogenates of lung, liver, spleen, heart and placenta (for fetuses) are listed for each mare that aborted. Mare D (*) did not abort, but lost a newborn foal. EHV-1 virus neutralisation titers (VNT) are specified for all mares in the barn

The first mare (A) aborted on February 12, 2017. The mare was immediately placed in strict isolation, and her stall in the main barn was thoroughly cleaned and disinfected. About a month later (March 9) two further mares (B and C) aborted within 2 days. On March 12, mare D gave birth to a weak foal with jaundice and respiratory disease that died 3 days later. Between March 16 and March 28 five further mares (E – I) aborted (Fig. 1). The aborting mares were reported to have a low-grade fever (38.5–39.5 °C) around the time of abortion by the stud’s veterinarian, but no other premonitory clinical signs. There were no serious clinical complications recorded after the expulsion of fetuses.

### Pathology

Complete necropsies were performed on eight aborted fetuses and the newborn foal by the attending veterinarian. All fetuses were fresh and enclosed within the fetal membranes. There were no apparent gross lesions in either the fetuses or placentas. Tissue samples (lung, liver, spleen, heart, and placenta) were submitted to the Department of Virology of the National Veterinary Research Institute in Pulawy (Poland) within 24 h of collection for laboratory investigation to identify the cause of abortion.

### Quantitative PCR

On arrival at the laboratory, 2.0 g of each tissue was homogenised with 18 mL of Eagle Minimal Essential Medium (Sigma-Aldrich) supplemented with 1% antibiotic solution (Sigma-Aldrich). Following a low-speed centrifugation, supernatants from tissues from the same fetus were pooled and stored at − 80 °C. DNA was extracted from 200 μL of each pooled supernatant using QIAamp DNA Mini Kit (Qiagen, Germany) according to the manufacturer’s recommendations and tested for the presence of EHV-1 and EHV-4 DNA using previously published primers from the conserved region of glycoprotein B [29, 30]. Positive (EHV-1 438/77 and EHV-4 405/76, American Type Culture Collection) and negative (water) controls were included in each PCR run. Samples were considered positive if the amplification curve crossed the threshold, as defined by automatic settings in the program (StepOne Plus™ Real-Time PCR System). All nine samples were positive for EHV-1, and none was positive for EHV-4 DNA.

### Virus isolation

Pooled supernatants from tissue homogenates were used for virus isolation in rabbit kidney 13 cells as described previously [25]. All samples developed viral cytopathic effect within 2 to 6 days post inoculation. The identity of the viral isolates was confirmed by EHV-1 specific PCR [29, 30].

### Genotyping and phylogenetic analysis

PCR assays to amplify fragments from ORF30 and ORF68 were performed as previously described [25]. A 380 bp ORF30 product was digested with *Sal*I in order to distinguish between N_752_ and D_752_ variants based on restriction fragment length polymorphism (RFLP) [25]. In addition, PCR products were sent for sequencing to Genomed S.A. (Poland). The sequences were assembled using the BioEdit software (version 7.2.5) and analysed using MEGA 5.0.5.

**Fig. 2:**
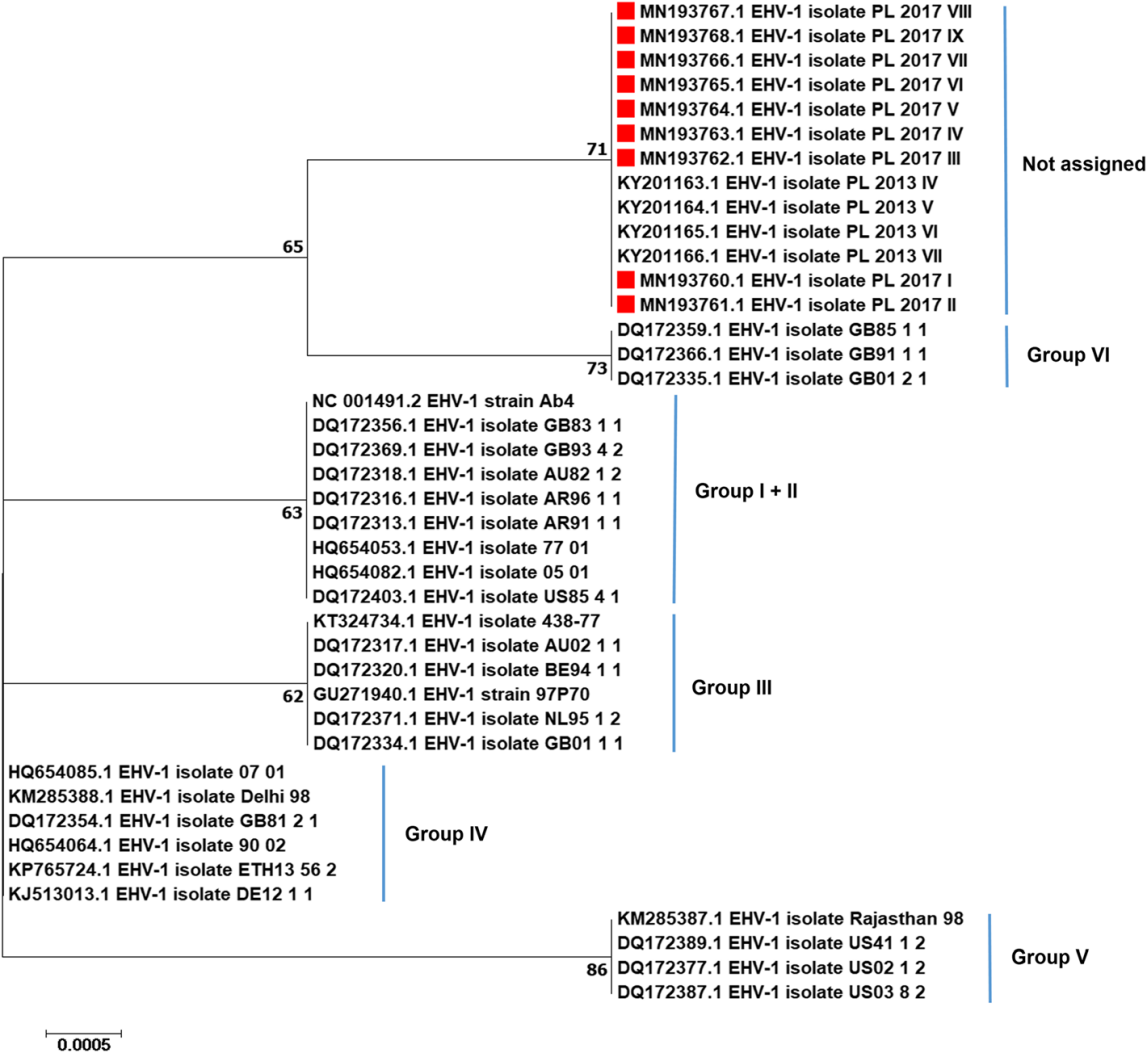
Phylogenetic tree of equid herpesvirus-1 based on 515 bp fragment from the ORF68 gene. The evolutionary history was inferred by using the Maximum Likelihood method based on the Kimura 2-parameter model [31]. The tree with the highest log likelihood (− 742.11) is shown. The percentage of trees in which the associated taxa clustered together is shown next to the branches. Initial tree(s) for the heuristic search were obtained automatically by applying Neighbor-Join and BioNJ algorithms to a matrix of pairwise distances estimated using the Maximum Composite Likelihood (MCL) approach, and then selecting the topology with superior log likelihood value. The tree is drawn to scale, with branch lengths measured in the number of substitutions per site. The analysis involved 41 nucleotide sequences including Polish EHV-1 sequences (*n* = 13) and international sequences (*n* = 28) sourced from GenBank (accession numbers included in the figure). All positions containing gaps and missing data were eliminated. There were a total of 495 positions in the final dataset. Evolutionary analyses were conducted in MEGA7 [32]. The clusters were labelled as groups I - VI according to [22]. The sequences obtained in the current study are labelled with red rectangles

Based on RFLP analysis of the ORF30 amplicon, all nine viruses from the stud belonged to ORF30 N_752_ genotype. This was further confirmed by sequencing. A phylogenetic tree of the 515-bp ORF68 gene fragment was reconstructed using the maximum likelihood method (ML) with 1000 bootstrap replicates using the Kimura 2-parameter model [31] in MEGA7 software [32] (Fig. 2). All nine ORF68 sequences were identical to each other and to four EHV-1 sequences (PL_2013_IV to VII) obtained from the abortion cases that occurred at the same stud in 2013 [25].

### GenBank accession numbers

The nucleotide sequences of Polish EHV-1 described in this study were submitted to GenBank under the following accession numbers: MN193769 - MN193777 (ORF30) and MN193760 - MN193768 (ORF68).

### Serology

Blood samples for serology were collected from all 30 mares in the barn once on April 18, 3 weeks after the last abortion case occurred. The presence of EHV-1 specific antibodies was determined using the virus neutralization test (VNT) according to the Manual of Diagnostic Tests and Vaccines for Terrestrial Animals protocol [33]. All serum samples were positive for EHV-1 antibody, with titers ranging from 8 to 256 (Fig. 1).

### Disease management and outcome

Following the first three abortion cases, the following procedures were introduced:

- Disinfection mats were placed in front of the entrance to each stable.
- All movement of horses to and from the stud was suspended. In addition, the stud was closed to outside visitors.
- Mares that aborted were isolated from other horses for a period of 30 days following abortion.
- Staff movement between stables was restricted and only designated people were allowed to care for pregnant mares.
- Immediate introduction of EHV-1 vaccination of all horses.
- Foaling staff was required to use disposable coveralls and gloves when handling mares and foals.
- All stalls, stables and equipment routinely used at the stud were thoroughly cleaned and disinfected.

All mares that aborted were successfully covered and produced healthy foals in the following (2017/18) breeding season.

## Discussion and conclusions

Equid herpesvirus 1 is one of the common causes of infectious abortion in horses [34]. Typical for EHV-1 abortions, all mares described in this report aborted in the last trimester of pregnancy. The reason for this timing is not fully understood, but it may be linked to an increased susceptibility of uterine endothelial cells to EHV-1 infection in late pregnancy [35]. The virus infection of endothelial cells of blood vessels in the gravid uterus leads to severe vasculitis and multifocal thrombosis, which are thought to be responsible for abortion [35–37]. The expelled fetuses were fresh, without apparent gross lesions, which is consistent with reports by others [11]. It is possible that mild gross lesions (if present) may have been missed, as the necropsies were performed by the stud’s veterinarian rather than a qualified pathologist. As the signalment was suggestive of EHV-1 involvement, virological investigation was instigated, which lead to detection of EHV-1 from tissues of aborted fetuses, and confirmed aetiological involvement of the virus.

The tissues used for virus detection/isolation included spleen, liver, lung, heart and placenta. The former three have been shown to contain the highest levels of the virus and are therefore considered to be preferred samples for testing [12]. Occasionally, abortion occurs due to placental insufficiency induced by virus infection before EHV-1 translocates to the fetus. On those rare occasions, the fetus may be virus-negative, which underscores the importance of testing placenta, if available [12].

Abortion storms are thought to result from horizontal transmission of EHV-1 between pregnant mares, but the initial source of the virus is often difficult to establish [2, 3]. It may be a newly introduced horse with an active EHV-1 infection, but equally it could be reactivated virus from one of the resident horses. The latter was a more likely source of the virus in the current outbreak for several reasons.

Firstly, the pregnant mares had been resident at the stud for years, managed by experienced handlers and were separated from young stock and other horses. Hence, they were kept under conditions that minimized stress. The triggers for EHV-1 reactivation from latency are poorly understood, but it is generally believed that stressful conditions such as unsettled social structure, changes in the daily routine, travelling or participation in competitive events are likely to play a role in that process [1, 38]. Secondly, it has been suggested that pregnant mares are predisposed to reactivation of EHV-1, which may be linked to physiological immunosuppression that is induced in the horse during pregnancy [39]. Lastly, the fact that all EHV-1 sequences obtained in the current study were identical to each other and to the four EHV-1 sequences obtained from the previous abortion cases at the same stud [25] also supports recrudescence of a latent infection, with the subsequent horizontal spread, as a likely source of the virus in the current outbreak.

Alternatively, EHV-1 with increased virulence may have been introduced to the stud from external sources during 2016/17 breeding season. We consider it less likely for several reasons. All horses were quarantined before introduction or re-introduction to the stud for a period of 2 weeks. Although EHV-1 DNA was occasionally detected in nasal secretions for up to 3 weeks following experimental infection [40], the majority of horses with active EHV-1 infection cease shedding the live virus within 1 to 2 weeks post infection [11, 17, 40–42]. Even if an occasional horse shed EHV-1 after being released from the quarantine, strict separation between pregnant mares and other horses should have minimized the chances of transmission of such virus to the mares.

Irrespective of the initial source of the virus, subsequent transmission of EHV-1 among pregnant mares led to the loss of pregnancies, including one death of a neonatal foal, for 9/30 pregnant mares. It is likely that the virus had been circulating among horses at the stud in prior seasons, without any clinically important consequences. Such pattern of EHV-1 circulation between vaccinated mares and their foals was described in several Australian-based studies [43–46]. One notable feature of the current outbreak is the fact that it coincided with the lapse in the routine yearly EHV-1/4 vaccination of the mares. This suggests that vaccination of mares in the prior breeding seasons induced protective immunity that reduced the likelihood of abortions. The occurrence of an abortion storm in the first season when mares were not vaccinated against EHV-1 underscores the fact that duration of immunity following EHV-1 vaccination is thought to be short [47]. It also highlights the importance of including vaccination as part of a herd-health program, as it limits the severity of clinical disease as well as duration and magnitude of EHV-1 shedding [48]. However, efficacy of currently available EHV-1 vaccines vary and none of them prevent establishment of EHV-1 infection or latency [46, 47]. Abortions can occur in the face of vaccination, as it happened at this stud in 2013, and has also been reported elsewhere [2, 46, 49]. Hence, vaccination should not be viewed as a substitute for good management practices including appropriate infection control measures.

All aborting mares described in this report showed mild fever at the time of abortion. This may have represented the first or second peak of biphasic fever typical for EHV-1 infections. The first peak can occur as early as 1 to 2 days post infection and is hence considered to be one of the most consistent early signs of EHV-1 infection both under field [5, 50, 51] and experimental [52] conditions. Consequently, implementation of temperature monitoring of all in-contact horses as early as possible in the outbreak situation is a common recommendation [38]. The febrile horses should be considered infectious and isolated from the rest of the population. Implementation of this control measure in the current outbreak may have minimised the number of abortion cases. It would have also provided some data on the infection status of the non-aborting mares, as blood samples or nasal swabs could have been collected from febrile horses for detection of the virus. Considering the close contact between mares at the turnout time, it is likely that most of the mares in the barn were infected with EHV-1, but only some aborted. This is supported by relatively high VNT titres (≥64) in 10/21 pregnant mares that did not abort. It is unlikely that such high titres represented residual titres from vaccination in the previous breeding season. Other control measures implemented at the stud including isolation of mares following abortions, control of traffic, use of personal protective equipment, hand washing, as well as cleaning and disinfection of stalls and equipment were likely to contribute to the control of the outbreak, consistent with recommendations of others [10, 50].

The VNT titres of the pregnant mares ranged from 8 to 256, without any apparent differences between the titres of the mares that aborted and those that have not. This underscores the limited value of the VNT in investigations of EHV-1 induced abortions. Paired serum samples were not collected, which is a limitation of the study. However, it is often difficult to detect a fourfold rise in VNT titres between acute and convalescent samples, as the antibody titres may have already risen by the time abortion occurs and acute samples are collected [2, 20, 53]. Determination of levels of complement fixing antibody may have been more useful as an indication of recent infection, as these decline sooner following EHV-1 infection or recrudescence than VN antibodies [47, 53]. Other problems related to EHV-1 VNT include cross-reaction with antibodies raised in response to EHV-4 infection [54], as well as lack of correlation between the levels of VN antibodies and protection from abortion [47]. Cell-mediated immune responses are believed to provide protective immunity [47, 55, 56], but assessment of such responses is complex and beyond the typical capabilities of a diagnostic laboratory.

All the viruses described in the current report belonged to the ORF30 N_752_ genotype, consistent with the predominance of the N_752_ variants among EHV-1 abortion cases in Poland [25, 57] and other countries [22, 28, 58]. While data from a recent investigation in Ireland [27] suggested that viruses with D_752_ genotype were more likely not only to induce neurological disease, but also to be associated with multiple abortion cases as opposed to sporadic abortions, our results underscore the fact that infection with viruses of either N_752_ or D_752_ genotype should be considered equally important, and similar precaution/control measures should be implemented to minimise the economic losses due to disease outcomes associated with these infections.

## Data Availability

The data sets supporting the results of this article are included within the article.

## Abbreviations

EHV-1: Equid herpesvirus 1
EHV-4: Equid herpesvirus 4
ORF30: Open reading frame 30
ORF68: Open reading frame 68
PCR: Polymerase chain reaction
RFLP: Restriction fragment length polymorphism
VNT: Virus neutralization test
